# Neutropenia and the Risk of Clostridioides difficile Infection, Fungemia, and Bacteremia: A Nationwide Inpatient Sample Analysis

**DOI:** 10.7759/cureus.114065

**Published:** 2026-08-06

**Authors:** Abdelrazig Suliman, Leonel Teran

**Affiliations:** 1 Internal Medicine, University of Texas at Tyler Health Science Center, Tyler, USA; 2 Internal Medicine, The University of Texas at Tyler, Tyler, USA

**Keywords:** bacteremia, c. difficile, fungemia, hcup, neutropenia, nis

## Abstract

Background

This study aimed to examine the association of neutropenia with *Clostridioides difficile* infection (CDI), fungal bloodstream infection, and bacteremia and to evaluate the unadjusted associations of these infections with in-hospital mortality among neutropenic hospitalizations.

Methodology

This retrospective cross-sectional study used data from the Healthcare Cost and Utilization Project Nationwide Inpatient Sample database from 2017 to 2020. Logistic regression models were applied to assess the association between neutropenia, infection rates, and the likelihood of in-hospital mortality.

Results

Patients with neutropenia were 3.69 times more likely to develop CDI (95% confidence interval (CI): 3.11-4.38), 7.08 times more likely to develop fungemia (95% CI: 5.27-9.73), and 5.07 times more likely to develop bacteremia (95% CI: 4.63-5.56). Among neutropenic patients, the odds ratio (OR) for fungemia was 3.21 (95% CI: 2.35-4.30, p < 0.001), whereas the OR for CDI was not statistically significant (p = 0.122). The OR for bacteremia was 0.60 (95% CI: 0.48-0.74, p < 0.001). This unadjusted result should not be seen as protective and is considered hypothesis-generating.

Conclusions

In this sample, neutropenia was associated with higher odds of all three infections, and fungemia was associated with higher in-hospital mortality. These results should be viewed with caution because the analyses were unweighted and unadjusted, and the database did not include detailed information on clinical severity or treatment.

## Introduction

Neutropenia is a blood disorder in which the number of neutrophils falls below normal, weakening the body’s natural immune defenses and increasing the risk of bacterial, fungal, and other infections [1,2]. This condition commonly affects people receiving chemotherapy, stem cell transplants, or immunosuppressive drugs, as well as those with blood cancers or serious chronic illnesses. Because neutrophils are a key component of the body’s first line of defense against infection, both the severity and duration of neutropenia are closely associated with an increased risk of infection and poorer health outcomes [1,2].

Despite advances in antibiotics, early detection, and supportive cancer care, infections remain a major cause of illness and death among people with neutropenia [3,4]. Bloodstream infections are especially serious in these patients because of weakened immune systems, frequent invasive procedures, prolonged hospital stays, damaged mucosal barriers, and increased exposure to antibiotics [3-5]. Research has shown that neutropenic patients have substantially higher rates of bacteremia caused by both Gram-positive and Gram-negative bacteria, including multidrug-resistant organisms. These infections are often associated with longer hospital stays, septic shock, and higher mortality rates [4-7].

Fungal bloodstream infections are also a major cause of death in individuals with weakened immune systems. Fungi such as *Candida* are especially dangerous for neutropenic patients because they are difficult to diagnose and treat, often leading to poor outcomes [8,9]. Studies have shown that invasive fungal infections are most common in patients with prolonged neutropenia and blood cancers, where damaged mucosal barriers and impaired immune function allow fungi to spread [9]. These infections often require greater hospital resources, more frequent intensive care admissions, and are associated with a higher risk of death [8].

*Clostridioides difficile* infection (CDI) is also an increasingly common problem among neutropenic and cancer patients. These patients are at higher risk for CDI because they are often hospitalized, receive broad-spectrum antibiotics, experience chemotherapy-related gastrointestinal injury, take acid-suppressive medications, and have disruptions in gut bacteria [10-13]. Studies have found that CDI can complicate care in patients receiving intensive chemotherapy or stem cell transplants, leading to longer hospital stays and increased strain on healthcare resources [10,12]. Alterations in the gut microbiome in immunocompromised patients increase susceptibility to both gastrointestinal and systemic infections [13].

Many studies in hospital settings or focused on specific diseases have examined infections in neutropenic patients, but there is little national-level data examining neutropenia, CDI, fungal bloodstream infection, and bacteremia together. Most research is limited to cancer centers, transplant patients, or small cohorts, making it difficult to generalize findings to all hospitalized adults in the United States [3,12]. In addition, the impact of these infections on mortality among neutropenic patients at the national level is not fully understood.

Hence, this study aimed to examine the association of neutropenia with CDI, fungal bloodstream infection, and bacteremia and to evaluate the unadjusted associations of these infections with in-hospital mortality among neutropenic hospitalizations.

## Materials and methods

This retrospective cross-sectional study used 2017-2020 data from the Healthcare Cost and Utilization Project (HCUP) National Inpatient Sample (NIS), sponsored by the Agency for Healthcare Research and Quality [14]. The NIS contains discharge-level records from a stratified sample of US community hospitals. All analyses used unweighted discharge records from the analytic sample. NIS discharge weights, sampling strata, and hospital-cluster variables were not incorporated; therefore, the reported estimates should not be interpreted as nationally weighted hospitalization estimates.

Neutropenia was identified using the International Classification of Diseases, 10th Revision, Clinical Modification (ICD-10-CM) code D70.9 (neutropenia, unspecified). CDI was identified using A04.71 (enterocolitis due to *C. difficile*, recurrent) and A04.72 (enterocolitis due to *C. difficile*, not specified as recurrent). Fungal bloodstream infection was the operational outcome label for B37.7. Bacteremia was identified using R78.81. These conditions were identified from diagnosis fields recorded for each hospitalization.

Study population

The study included hospitalized adults aged 18 years or older. Patients with neutropenia were identified using ICD-10-CM diagnosis codes for neutropenic disorders recorded during their hospital stay. Patients were then grouped into neutropenic and non-neutropenic cohorts. A random sample of non-neutropenic hospitalizations was selected to create a comparison group of approximately equal size. This sampling step balanced group size but did not match hospitalizations on demographic, clinical, or hospital characteristics.

Outcome variables

The primary outcomes of interest were the prevalence of CDI, fungemia, and bacteremia. These infectious complications were identified using ICD-10-CM diagnosis codes recorded during hospitalization. The secondary outcome was in-hospital mortality among neutropenic patients with CDI, fungemia, or bacteremia. Mortality status was identified using the HCUP discharge disposition variable indicating death during hospitalization.

Descriptive variables

The data on demographics covered age, sex, and race or ethnicity. Age was treated as a continuous variable and presented as mean and standard deviation. For the categorical variables, the frequencies and percentages were provided. These variables were included solely to describe the sample and were not used as covariates in the regression models.

Statistical analysis

Descriptive statistics were used to summarize demographic characteristics in the neutropenic and non-neutropenic groups. Continuous variables were reported using means and standard deviations, and medians with ranges when appropriate. Categorical variables were reported as frequencies and percentages. No hypothesis tests were performed for the descriptive comparisons.

Separate unadjusted, unweighted binary logistic regression models evaluated the association between neutropenia and each infection outcome, i.e., CDI, fungal bloodstream infection, and bacteremia. Odds ratios (ORs) with 95% confidence intervals (CIs) were calculated.

Within the neutropenic cohort, separate unadjusted, unweighted binary logistic regression models evaluated the association of CDI, fungal bloodstream infection, and bacteremia with in-hospital mortality. ORs with 95% CIs were reported. Two-sided Wald tests were used to evaluate the regression coefficients, and the corresponding Wald z-statistics are reported with the p-values. A two-sided p-value <0.05 was considered statistically significant.

Data cleaning, management, statistical analyses, and visualization were performed using R statistical software version 4.3.1 (RStudio, Vienna, Austria) [15].

## Results

Patient characteristics

The final analysis included 161,365 adult hospitalizations: 80,740 involved patients with neutropenia, and 80,625 involved patients without neutropenia (Table 1).

**Table 1:** Age, race, and gender by neutropenia versus non-neutropenia. Note. A random sample was drawn from the non-neutropenic hospitalizations in the full dataset to create a comparison group of approximately equal size. The groups were not matched on patient or hospital characteristics.

	Neutropenia: No	Neutropenia: Yes
	(N = 80,625)	(N = 80,740)
Age in years at admission		
Mean (SD)	58.2 (20.2)	59.6 (16.8)
Median (minimum, maximum)	61.0 (18.0, 90.0)	62.0 (18.0, 90.0)
Gender		
Male	34,649 (43.0%)	39,785 (49.3%)
Female	45,965 (57.0%)	40,950 (50.7%)
Missing	11 (0.0%)	5 (0.0%)
Race		
White	52,284 (64.8%)	52,142 (64.6%)
Black	12,049 (14.9%)	11,940 (14.8%)
Hispanic	8,956 (11.1%)	8,649 (10.7%)
Asian or Pacific Islander	2,172 (2.7%)	2,834 (3.5%)
Native American	497 (0.6%)	406 (0.5%)
Other	2,457 (3.0%)	2,659 (3.3%)
Missing	2,210 (2.7%)	2,110 (2.6%)

Neutropenic patients were slightly older than those without neutropenia, with a mean age of 59.6 years compared with 58.2 years. The median age was also slightly higher among neutropenic patients (62 years versus 61 years). Men accounted for a larger proportion of the neutropenic group (49.3% versus 43.0%), while women accounted for 50.7% of neutropenic patients and 57.0% of non-neutropenic patients.

Most patients in both groups were White, accounting for 64.6% of neutropenic patients and 64.8% of non-neutropenic patients. Black patients accounted for 14.8% and 14.9% of the two groups, respectively, while Hispanic patients accounted for 10.7% and 11.1%, respectively. Fewer patients were Asian or Pacific Islander, Native American, or from other racial backgrounds. Overall, the groups had similar demographic profiles.

Prevalence of infectious complications

CDI was more common in neutropenic patients than in those without neutropenia (Table 2). It occurred in 617 (0.76%) neutropenic hospitalizations and 168 (0.21%) non-neutropenic hospitalizations. Neutropenic patients had 3.69 times higher odds of CDI (OR = 3.69, 95% CI: 3.11-4.38; Wald z = 14.95, p < 0.001).

**Table 2:** Prevalence of Clostridioides difficile, fungemia, and bacteremia in neutropenic patients. Note: Unadjusted ORs were estimated using separate unweighted binary logistic regression models. The p-values are from two-sided Wald tests of the regression coefficients; the corresponding Wald z-statistics are shown. CDI = *Clostridioides difficile* infection; CI = confidence interval; OR = odds ratio

	Neutropenia: No	Neutropenia: Yes	OR	P-value	Wald z
	N = 80,625	N = 80,740			
CDI					
No	80,457 (99.8%)	80,123 (99.2%)	Ref.	Ref.	
Yes	168 (0.21%)	617 (0.76%)	3.69 (3.11, 4.38)	<0.001	14.95
Fungemia					
No	80,578 (99.9%)	80,407 (99.6%)	Ref.	Ref.	
Yes	47 (0.06%)	333 (0.41%)	7.08 (5.27, 9.73)	<0.001	12.51
Bacteremia					
No	80,068 (99.3%)	77,991 (96.6%)	Ref.	Ref.	
Yes	557 (0.69%)	2749 (3.40%)	5.07 (4.63, 5.56)	<0.001	34.77

Fungemia was much more frequent in neutropenic patients, occurring in 333 (0.41%) neutropenic hospitalizations and 47 (0.06%) non-neutropenic hospitalizations. Neutropenia was associated with higher odds of fungemia (OR = 7.08, 95% CI: 5.27-9.73; Wald z = 12.51, p < 0.001).

Bacteremia was also more common in neutropenic patients, occurring in 2,749 (3.40%) neutropenic hospitalizations and 557 (0.69%) non-neutropenic hospitalizations. Neutropenic patients had 5.07 times higher odds of bacteremia (OR = 5.07, 95% CI: 4.63-5.56; Wald z = 34.77, p < 0.001).

**Figure 1:**
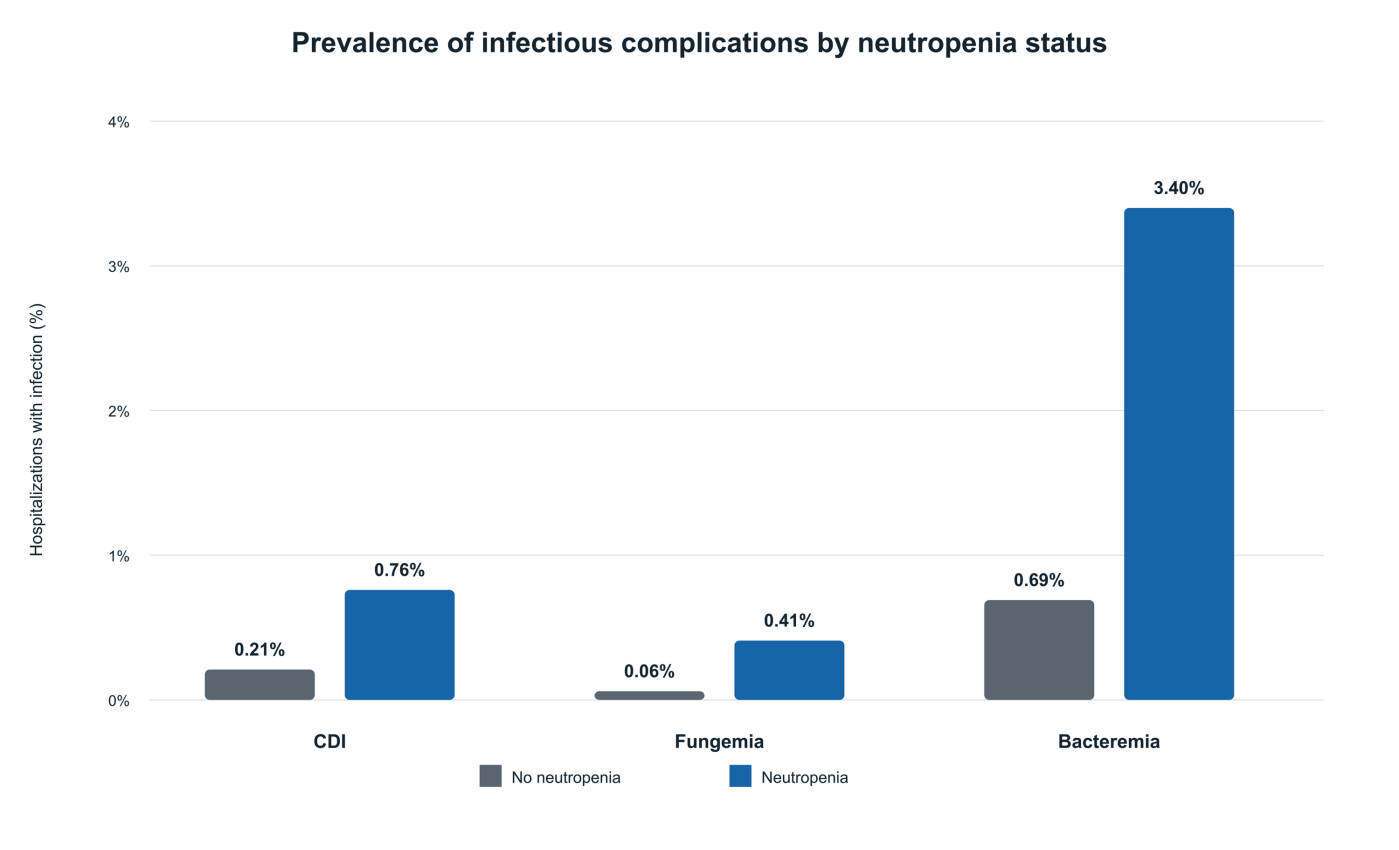
Differences in infection prevalence according to neutropenia status. Bars represent the percentage of hospitalizations with each infectious complication in the neutropenic and non-neutropenic groups. Fungal bloodstream infection was operationally identified using ICD-10-CM code B37.7. Results are based on the unweighted analytic sample. CDI = *Clostridioides difficile* infection; ICD-10-CM = International Classification of Diseases, 10th Revision, Clinical Modification

**Figure 2:**
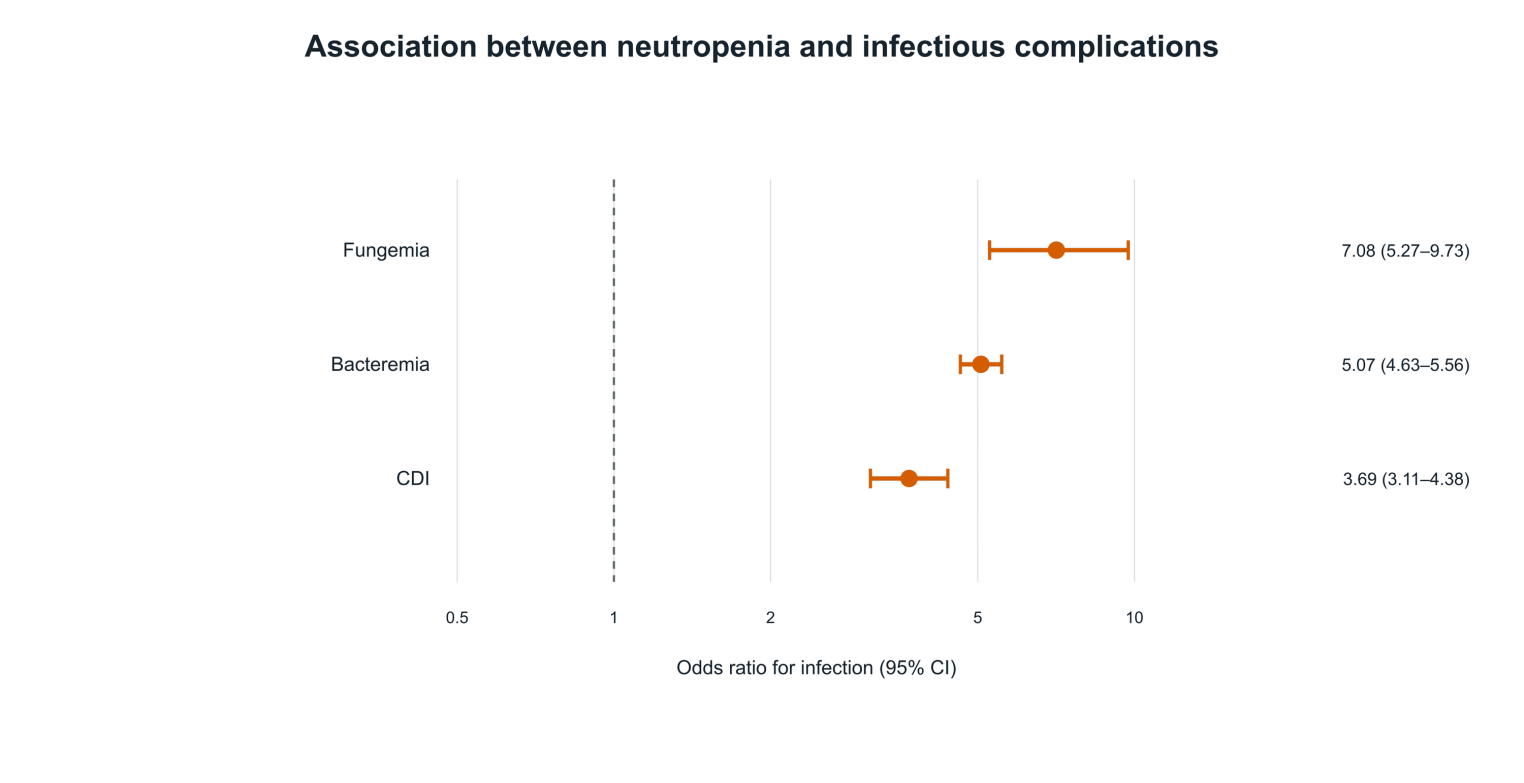
Association between neutropenia and infectious complications. Points represent unadjusted ORs, and horizontal error bars represent 95% CIs from separate unweighted binary logistic regression models. The dashed vertical line at an OR of 1 indicates no association. Values greater than 1 indicate higher odds of the infectious complication among hospitalizations involving neutropenia. Fungal bloodstream infection was operationally identified using ICD-10-CM code B37.7. CDI = *Clostridioides difficile* infection; ICD-10-CM = International Classification of Diseases, 10th Revision, Clinical Modification; OR = odds ratio; CI = confidence interval

Among the infections studied, fungemia showed the strongest association with neutropenia, followed by bacteremia and CDI. The differences in infection prevalence according to neutropenia status are shown in Figure 1. The corresponding unadjusted ORs for these infectious complications are summarized in Figure 2.

In-hospital mortality

In the neutropenic group, CDI was associated with higher odds of in-hospital death; however, this result was not statistically significant. The OR for in-hospital mortality among neutropenic patients with CDI was 1.29 (95% CI: 0.92-1.74; Wald z = 1.55, p = 0.122) compared with those without CDI (Table 3).

**Table 3:** Associations of Clostridioides difficile infection, fungemia, and bacteremia with in-hospital mortality among neutropenic patients. Note: Unadjusted ORs were estimated using separate unweighted binary logistic regression models. The p-values are from two-sided Wald tests of the regression coefficients; the corresponding Wald z statistics are shown. CDI = *Clostridioides difficile* infection; CI = confidence interval; OR = odds ratio

Infection	OR	95% CI	Wald z	P-value
CDI	1.29	0.92–1.74	1.55	0.122
Fungemia	3.21	2.35–4.30	7.57	<0.001
Bacteremia	0.60	0.48–0.74	−4.63	<0.001

In contrast, fungemia was strongly associated with higher odds of in-hospital death among neutropenic patients. Patients with fungemia had 3.21 times higher odds of in-hospital mortality compared with neutropenic patients without fungemia (OR = 3.21, 95% CI: 2.35-4.30; Wald z = 7.57, p < 0.001) (Table 3).

**Figure 3:**
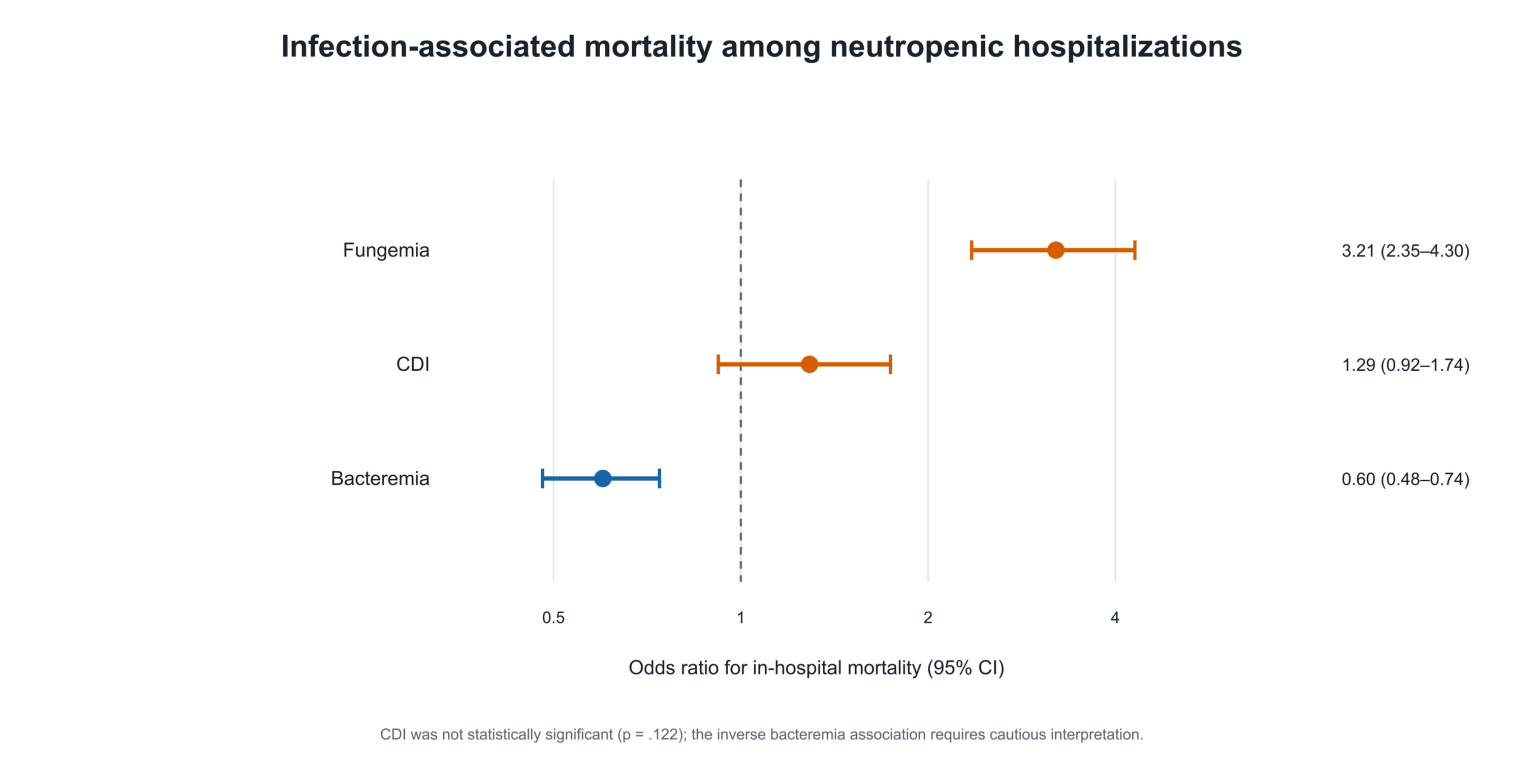
Infection-associated in-hospital mortality among neutropenic hospitalizations. Points represent unadjusted ORs, and horizontal error bars represent 95% CIs from separate unweighted binary logistic regression models. The dashed vertical line at an OR of 1 indicates no association. CDI was not significantly associated with in-hospital mortality. The inverse association between bacteremia and mortality was unexpected and should not be interpreted as evidence of a protective effect. Fungal bloodstream infection was operationally identified using ICD-10-CM code B37.7. CDI = *Clostridioides difficile* infection; ICD-10-CM = International Classification of Diseases, 10th Revision, Clinical Modification; OR = odds ratio; CI = confidence interval

Bacteremia was unexpectedly associated with lower odds of in-hospital death among neutropenic hospitalizations (OR = 0.60, 95% CI: 0.48-0.74; Wald z = −4.63, p < 0.001) (Table 3). This unexpected unadjusted association should not be interpreted as evidence of a protective effect. The mortality associations for all three infections are summarized in Figure 3.

Summary of findings

In this analytic sample, neutropenia was associated with higher odds of CDI, fungemia, and bacteremia. The strongest association was observed for fungal bloodstream infection, which was also associated with higher odds of in-hospital mortality among neutropenic hospitalizations.

## Discussion

In this large, multiyear, analytic NIS sample, neutropenia was associated with higher odds of CDI, fungal bloodstream infection, and bacteremia [1,3,4]. Fungal bloodstream infection showed the strongest association with neutropenia and was also associated with substantially higher odds of in-hospital mortality [8,9]. These findings reinforce that infectious complications remain an important clinical concern among hospitalized patients with neutropenia.

Neutrophils are essential to the body’s first line of defense, helping to recognize and destroy harmful microbes. When neutrophil counts decrease, the body becomes more vulnerable to bacterial and fungal infections [1,2]. Previous research has shown that individuals with neutropenia, particularly those with cancer or undergoing chemotherapy, have higher rates of bloodstream infections and sepsis [3,4]. The associations observed in this analytic sample are consistent with that literature.

The link between neutropenia and bacteremia is supported by both biological and clinical mechanisms. Neutropenic patients often experience disruption of mucosal barriers due to chemotherapy, prolonged hospital stays, central venous catheters, repeated procedures, and extensive antibiotic use, all of which increase the risk of bacterial entry into the bloodstream [3,4,6]. Studies have also shown shifts in bacterial profiles and increasing antibiotic resistance in these patients [4,6,7]. Our finding of more than five times higher odds of bacteremia underscores the need for early detection, prompt antibiotic therapy, and close infection monitoring in patients with neutropenia.

Fungal bloodstream infection showed the strongest association with neutropenia and was also associated with substantially higher odds of in-hospital mortality. This finding is consistent with previous research showing that invasive fungal infections can be particularly severe in immunocompromised patients [8,9]. Fungal infections, especially those caused by *Candida* species, remain a major cause of illness and death in patients with prolonged neutropenia, hematologic malignancies, or stem cell transplantation [9]. Because this study was observational and unadjusted, the findings support clinical vigilance but do not establish that any specific intervention improves outcomes.

Our analysis also demonstrated significantly increased odds of CDI among neutropenic patients. These findings are consistent with previous investigations showing that immunocompromised individuals, particularly those with cancer, are at higher risk of CDI due to frequent antibiotic use, chemotherapy-related gut damage, prolonged hospital stays, acid-suppressive medications, and alterations in gut bacteria [10-13]. Changes in the gut microbiome are now recognized as an important factor contributing to increased susceptibility to both gastrointestinal and systemic infections in these patients [13]. While CDI was more common in neutropenic patients in our study, it was not significantly associated with an increase in in-hospital mortality. Additionally, CDI severity may vary considerably among hospitalized patients, and administrative datasets may not fully capture disease severity, recurrence, or treatment timing. Prior studies in hematopoietic stem cell transplant populations have similarly shown that, although CDI increases healthcare utilization and complicates hospitalization, its effect on mortality may vary depending on comorbidities and underlying disease burden [10,12].

The finding that bacteremia was associated with lower in-hospital mortality was surprising and unexpected and does not align with what is typically observed in clinical studies. As this result came from an unadjusted model, it should not be taken as proof that bacteremia is protective or leads to better survival on its own. This link could be due to factors such as unmeasured confounding, differences in patients’ underlying conditions and illness severity, selection bias, coding differences, how bacteremia is defined, treatment factors, or other limitations of using administrative data. This result should be seen as a starting point for further research and needs to be confirmed in studies that use detailed clinical, microbiological, and treatment data.

This study has several strengths. It used a large, multiyear sample of inpatient discharge records and examined several clinically important infectious outcomes within the same analytic cohort. The sample size also permitted evaluation of the relatively uncommon outcome of fungal bloodstream infection. However, because survey weights were not used, these strengths should not be interpreted as establishing nationally weighted estimates.

There are several important limitations to note. Because this was a retrospective cross-sectional study, we cannot determine causality or the order in which diagnoses occurred. Using ICD-10-CM codes could lead to misclassification, and we defined fungal bloodstream infection (fungemia) using code B37.7. As our analyses were unweighted, the results should not be seen as nationally representative. The regression models were not adjusted for factors such as demographics, underlying cancer, other health conditions, transplants, illness severity, or hospital characteristics. The NIS database also does not include information such as absolute neutrophil counts, the duration or severity of neutropenia, details about chemotherapy, antimicrobial or antifungal treatments, specific microbiology results, the timing of diagnosis and treatment, or detailed measures of how sick patients were. Because of these gaps, there may be significant confounding that we could not measure, especially regarding the unexpected link between bacteremia and mortality.

Even with these limitations, the study found some associations that deserve more research. In this group, neutropenia was linked to higher chances of CDI, fungal bloodstream infection, and bacteremia. Fungal bloodstream infection was also linked to a higher risk of dying in the hospital. Future studies should adjust for clinical severity, microbiology, treatment, and timing to better understand these relationships.

## Conclusions

In the unweighted analysis, neutropenia was linked to higher odds of CDI, fungemia, and bacteremia. Fungemia had the strongest association with neutropenia and was also linked to higher in-hospital mortality. These results show associations, not causality, and should not be considered nationally representative. These findings underscore the importance of vigilant infection surveillance in hospitalized patients with neutropenia. However, as this analysis was unadjusted, it does not establish whether specific monitoring or treatment strategies improve patient outcomes. The unexpected association between bacteremia and lower mortality should not inform clinical decision-making or be interpreted as a protective effect. This finding is intended to generate hypotheses for future research and may reflect confounding factors, selection bias, coding inaccuracies, or unmeasured differences in illness severity and treatment. Future research should employ adjusted models and incorporate variables such as absolute neutrophil counts, severity and duration of neutropenia, comorbidities, cancer type, microbiological data, antibiotic use, illness severity, hospital characteristics, and timing of diagnosis and treatment.
